# Ultrasonographic features of epididymitis in dogs: a case series

**DOI:** 10.3389/fvets.2025.1691917

**Published:** 2025-10-24

**Authors:** Stefano Spada, Marco Russo, Sebastian P. Arlt, Bianca L. Frehner, Gaia Pagani, Joana Rodrigues Carvalho, Hélène Jainek, Nicola Ambrosio, Daniela De Felice, Johannes Herbel

**Affiliations:** ^1^Clinic of Reproductive Medicine, Vetsuisse Faculty of Zurich, University of Zurich, Zürich, Switzerland; ^2^Department of Veterinary Medicine and Animal Production, University of Naples Federico II, Naples, Italy

**Keywords:** epididymitis, ultrasound, dog, testicles, inflammation

## Abstract

**Introduction:**

Epididymitis in dogs is an underreported but clinically relevant condition, often associated with scrotal pain, systemic inflammation, and infertility. Diagnosis commonly relies on clinical and ultrasonographic findings, even though information concerning the latter are poorly documented. This report aims to describe the ultrasonographic features of epididymitis in a series of clinical canine cases, including clinical and laboratory findings.

**Materials and methods:**

Clinical cases of dogs affected by epididymitis were retrospectively included. Clinical signs, andrological findings, ultrasonographic abnormalities of the epididymis and testicles, blood analysis and, where available, cytological or histopathological data were recorded. Ultrasonographic features evaluated included epididymal size, capsular integrity, echotexture, vascularization, presence of mineralization and scrotal effusion.

**Results:**

Fourteen dogs with epididymitis were included, and the condition was unilateral in 11 dogs (eight left-sided) and bilateral in three. On B-mode ultrasound the epididymal tail was the most frequently affected portion. Other frequent findings included epididymal enlargement, capsular irregularity, and inhomogeneous echotexture. Mineralization, hypoechoic cavities, and peri-epididymal oedema were observed, but not present in all cases. Concurrent testicular and urogenital abnormalities, along with leucocytosis and neutrophilic left shift, were common in acute cases.

**Conclusion:**

This is the first case series describing ultrasonographic patterns of epididymitis in dogs. Early recognition via ultrasound may support targeted treatment and fertility preservation.

## 1 Introduction

Epididymitis is an inflammation of the epididymis that occurs across most domestic species and may have an infectious or non-infectious origin (1–3). Among infectious agents, *Brucella* spp. is the most well-known pathogen inducing epididymitis in dogs (4), even though other bacterial species have also been isolated, such as *β-hemolytic Streptococcus* (1), *Escherichia coli* (5), and *Salmonella* spp. (6). The disease can be the result of ascending infections originating from other organs of the urogenital tract or haematogenous spread (1). In dogs, epididymitis can be unilateral or bilateral and occur alone, or in association with orchitis (orchi-epididymitis), or involving the vaginal tunic as well, generating scrotal adhesions (periorchi-epididymitis) (1). The presentation may be clinically relevant for prognosis and treatment decisions, especially, in unilateral cases, where hemicastration may be considered to preserve future fertility in breeding dogs (7). Epididymitis can be either acute or chronic, with the acute stage being typically associated with more severe clinical signs such as scrotal pain and redness, lameness, lethargy and purulent urethral discharge (5). In severe cases, the condition can evolve into sepsis posing a life-threatening risk (1). Thus, early diagnosis and intervention are crucial for improving prognosis, minimizing recovery time and preserving reproductive function. In contrast, chronic cases may manifest as scrotal and epididymal enlargement without pain or discomfort (5), although atrophy and fibrosis are common long-term outcomes (7).

Diagnostic approaches include clinical examination, bacterial culture and cytological examination of the ejaculate or scrotal effusion, if present, as well as ultrasonography (7). However, it should be emphasized that semen collection can be challenging in dogs with inflammatory processes of the urogenital tract due to the pain occurring during stimulation (8). Ultrasound represents the method of choice to assess testicular and epididymal parenchyma and to detect potential abnormalities (9). Anatomically, each epididymis consists of three different portions: head, body and tail, located cranially, dorsally and caudally to the testicle, respectively (10). The tail continues as the ductus deferens, which enters the abdominal cavity via the inguinal canal and connects to the urethra (11). On B-mode ultrasound, the epididymis typically appears hypoechoic when compared to the ipsilateral testicle. When imaged laterally and longitudinally, the tail and the head exhibit a normal and reversed capital “D” shape, respectively, located caudally and cranially to the testicle. The body is seen as an elongated dorsal structure (12–15). Despite its important role in andrological examination, the ultrasonographic appearance of normal and abnormal epididymal structures is still poorly documented in veterinary literature, especially in pathologic conditions, such as epididymitis (1, 4, 5, 16). Detecting early signs of inflammation or identifying predisposing factors may support clinical decision-making, particularly in dogs intended for breeding. The aim of this case series is to report and describe the specific ultrasonographic features of epididymitis in dogs.

## 2 Materials and methods

The present retrospective duo-centre case study included clinical cases of dogs diagnosed with epididymitis. Cases were collected consecutively from 2020 to 2025, from the clinical medical records of two institutions: the Veterinary Teaching Hospital of the Department of Veterinary Medicine and Animal Production, University of Naples, Federico II, and the Clinic of Reproductive Medicine, Vetsuisse Faculty of Zurich, University of Zurich.

Inclusion criteria were as follows:

- Clinical signs suggestive of epididymitis such as scrotal swelling, redness, hyperthermia, inflammation or pain;
- Ultrasonographic findings consistent with epididymal inflammation or fibrosis;
- Blood analysis findings supporting the differentiation between acute and chronic cases.

Dogs with incomplete records were excluded from the present study. Since histopathological examination of the epididymis was not available for all cases, dogs were classified as having presumptive acute or chronic epididymitis based on clinical signs, ultrasonographic findings and duration of disease.

Cases were classified as acute due to recent onset (< 2 weeks) of local or systemic inflammation (e.g., testicular swelling, hyperthermia, redness, pain), supported by ultrasonographic (e.g., scrotal fluid accumulation) and haematological evidence of acute inflammation (e.g., increased band neutrophiles, monocytosis, leucocytosis), and, when available, histopathological or cytological confirmation of an infection (e.g., presence of increased amount of neutrophiles, bacteria or necrosis, but without any signs of fibrotic process).

Chronic cases were defined by asymptomatic longstanding (> 2 weeks) epididymal abnormalities without current acute inflammatory signs, ultrasonographic evidence of fibrosis or reduced epididymal size, absence of active infection on imaging and hematology and, when available, histopathological or cytological confirmation of fibrotic processes affecting the epididymis.

Medical records were reviewed using a standardized data collection form.

A complete anamnesis and information concerning general and andrological examination, including evaluation of the penis and prepuce, inspection and palpation of the scrotum and testicles and digital rectal examination of the prostate gland, of all dogs were collected.

Ultrasonographic examinations were performed using two different machines (Logiq S8, GE Healthcare; DC90, Mindray) equipped with either linear (11–18 MHz) or microconvex (3–10 MHz) probes, with the choice of probe depending on the animal and testicular size. Image settings (e.g., gain, depth, focal zone, dynamic range) varied between examinations and operators. All examinations were carried out by experienced clinicians specialized in small animal reproductive imaging.

On B-mode ultrasound, size, shape, margins, echogenicity and echotexture were assessed and were considered normal based on the description provided by Mattoon and Davidson (13).

Each portion of the epididymis, including tail, body and head thickness (dorsoventral length) were measured from a lateral longitudinal scan and compared with contralateral epididymis to assess the location of the inflammation. The interpretation was performed using the reference ranges provided by Pugh et al. (12):

- Tail: 0.6–1.3 cm;
- Head: 0.4–0.8 cm;
- Body: 0.2–0.7 cm;

The affected epididymal portion and the presence of abnormal ultrasonographic features including lack of capsular integrity, presence of peri-epididymal oedema or scrotal effusion, mineralization, cysts, increased scrotal thickness and coexisting testicular abnormalities were recorded.

Vascularization was assessed when available, using one or more of the following techniques: Colour Doppler, Spectral Doppler, Pulsed Doppler, and Contrast-Enhanced Ultrasound (CEUS). The regions of interest included the pampiniform plexus and the epididymal parenchyma. Due to the retrospective nature of the case study and the lack of standardization across examinations (different ultrasound machines, probes, operators, settings, and sampling regions), quantitative comparisons between cases could not be performed. Therefore, only a subjective evaluation of vascularization was undertaken.

In particular, for Colour Doppler and Pulsed Doppler examinations, the vascular blood supply in the affected epididymis was subjectively assessed and, in unilateral cases, compared with the contralateral normal epididymis to detect relative increased vascularization suggestive of active inflammation. For CEUS examinations, contrast enhancement was subjectively evaluated, with specific attention to the intensity of enhancement, wash-in timing, and the degree of homogeneity of the enhancement pattern.

Findings from other organs such as the urinary bladder, prostate, ductus deferens and iliac lymph nodes, were recorded in order to detect potential concurrent urogenital involvement. Reference values for ductus deferens thickness (11), and iliac lymph nodes (17, 18) were used.

Blood analysis and cytology or histopathology (if present) results were documented. Since blood analyses were performed on different machines and across different laboratories, direct numerical comparison between cases was not feasible. Therefore, interpretation of results was limited to whether values were within, above, or below the respective reference intervals provided by the analyser used for each patient.

When present, cytology and histopathology were used to confirm the diagnosis and to assess the presence of signs of parenchymal chronicity.

Ethical review and approval were not required for this study because it was a retrospective analysis of anonymized clinical data obtained during routine diagnostic procedures. No experimental interventions were performed on client-owned animals, and written informed consent was not required in accordance with institutional and national guidelines.

Descriptive statistics (median, interquartile range) were used to summarize patient demographics and findings. Outcome measures such as resolution, progression to chronic disease, or castration were recorded when available, but were not used as inclusion or exclusion criteria.

## 3 Results

### 3.1 Animals

A total of 14 intact male dogs, with a median age of 10 years (IQR = 5.25–13) and a median body weight of 15 kg (IQR = 7.5–26.75) met the inclusion criteria. The dogs recruited represented various breeds, including Chihuahua (2), Labrador (1), Schnauzer (1), Chow-Chow (1), Boston Terrier (1), Bernese Mountain dog (1), Continental Bulldog (1), Spitz (1) and mixed breed dogs (5).

Epididymitis was unilateral in 11 cases and bilateral in three. Among the unilateral cases, eight involved the left epididymis and three the right. Based on anamnesis, clinical signs, ultrasonographic findings and histopathological confirmation, 10 cases were classified as acute epididymitis and four as chronic.

Blood analyses, comprehensive of blood cells count and biochemistry profile, were present in all dogs. Histopathological/Cytological examination was present in seven dogs confirming the presence of an epididymitis.

### 3.2 Anamnesis and clinical signs

Clinical and anamnestic findings varied between acute and chronic cases. Dogs identified with acute epididymitis showed clinical signs from 1 to 14 days prior to presentation, generally including apathy, lethargy and inappetence. In contrast, two chronic cases were clinically asymptomatic and the other two were presented with testicular enlargement but without any pain or discomfort.

A detailed summary of the clinical signs observed in the present population is reported in Table 1.

**Table 1:** Summary of the clinical signs observed in the present population.

Clinical sign	Number of cases (*n =* 14)	Acute (*n =* 10)	Chronic (*n =* 4)
Scrotal swelling	12	10	2
Scrotal pain	10	10	0
Scrotal redness/hyperthermia	4	4	0
Fever	3	3	0
Apathy/lethargy/inappetence	11	10	1
Urogenital signs	8	6	2
Gastrointestinal signs	4	3	1
No clinical signs	2	0	2

The most frequent clinical signs across all enrolled dogs were scrotal swelling and scrotal pain. Less common findings included scrotal redness and hyperthermia, and systemic symptoms such as fever or gastrointestinal disturbances (vomiting and diarrhoea). Signs of lower urinary tract diseases like incontinence, haematuria or urinary stones were noted in both acute and chronic cases.

Interestingly, in two chronic cases, epididymis appeared normal or reduced in size, and the inflammatory process was detected incidentally during routine ultrasound examination. Both dogs had a history of urogenital diseases, i.e., in one dog a prior scrotal hernia which was surgically repaired 5 months before presentation and in the other dog a recurrent cystitis.

### 3.3 Ultrasound examination

**Figure 1:**
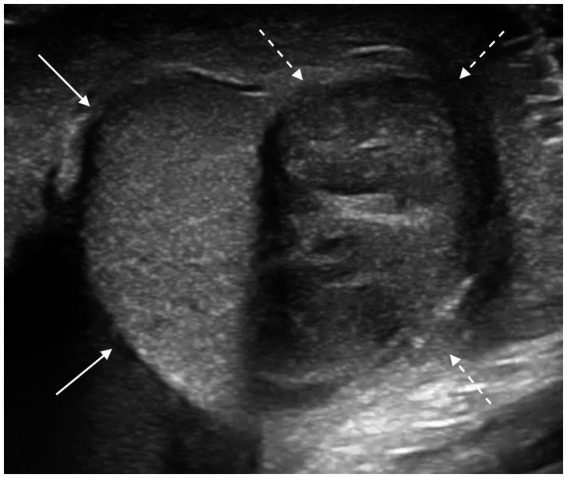
Transverse scan of acute epididymitis affecting the body of the epididymis (white dashed arrows), which appears enlarged, nearly matching the thickness of the ipsilateral testicle (white solid arrows), with regular margins and inhomogeneous echotexture.

**Figure 2:**
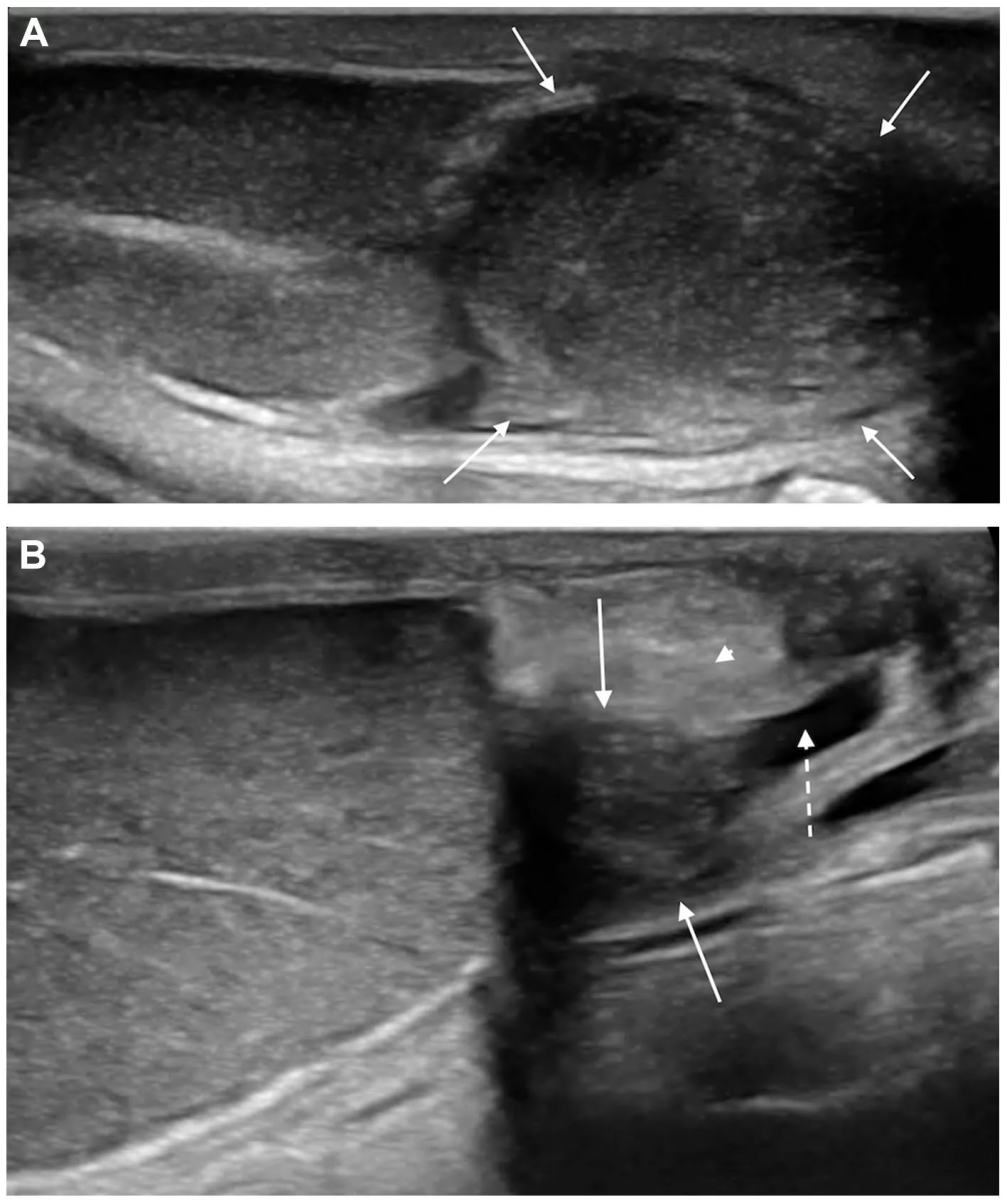
Longitudinal scan of an acute **(A)** and chronic **(B)** epididymitis of the tail. **(A)** In the acute form the epididymal tail (white solid arrows) is enlarged, rounded, with well-defined margins and inhomogeneous echotexture. **(B)** The chronic form shows a reduced epididymal tail (white solid arrows) with irregular margins, mild inhomogeneity of the parenchyma, hyperechogenicity of the vaginal tunic (white arrowhead), and anechoic scrotal effusion (white dashed arrow).

In nine cases, the entire epididymis (head, body, and tail) was affected, whereas in three cases, involvement was limited to the tail and body. In two cases, only the tail was affected. All acute cases showed epididymal enlargement (Figure 1), particularly evident in unilateral presentations when compared to the contralateral side. Among chronic cases, two dogs exhibited enlargement, while the remaining two had normal and reduced epididymal dimensions (Figure 2). Sizes of normal and abnormal epididymis are reported in Table 2.

**Table 2:** Median and interquartile ranges of normal and abnormal epididymis found in the present population.

Epididymal portion	Normal epididymis	Abnormal epididymis
Head (cm)	0.7 (0.45–0.8)	1.4 (1.05–1.85)
Body (cm)	0.3 (0.2–0.69)	1.6 (1.12–2.07)
Tail (cm)	0.8 (0.6–0.95)	1.53 (1.32–2.25)

**Figure 3:**
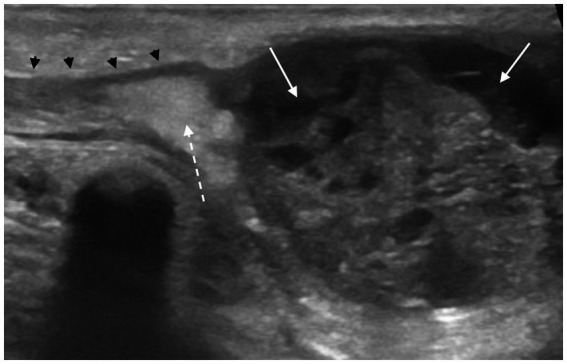
Longitudinal scan of acute epididymitis localized to the head of the epididymis. The affected region shows increased size, irregular margins, mixed echogenicity, and inhomogeneous echotexture due to multiple cavities/abscesses (white solid arrows). Increased echogenicity of the peritesticular fat (white dashed arrow), suggestive of steatitis could be observed in the area of the caudal portion of the ductus deferens (black arrowheads).

Ultrasonographic signs affecting the epididymis are summarized in Table 3. Both acute and chronic cases exhibited capsular irregularity and inhomogeneous echotexture, often with parenchymal hyperechoic stippling. Other additional findings were hypoechoic cysts suggestive of abscessation, mineralization, peri-testicular or peri-epididymal oedema and scrotal effusion. In particular, the latter was noted in three dogs, being anechoic in two cases (suggestive of hydrocele), and echogenic in one (suggestive of pyocele) (Figure 3).

**Table 3:** Summary of the ultrasonographic findings of the epididymis observed in the present population.

Ultrasonographic sign	Number of cases (*n =* 14)	Acute (*n =* 10)	Chronic (*n =* 4)
Epididymal enlargement	12	10	2
Capsular irregularity	14	10	4
Inhomogeneous echotexture	14	10	4
Hypoechoic cavities (abscess/cysts)	7	6	1
Mineralization	8	5	3
Peri-epididymal oedema	3	2	1
Scrotal effusion	3	2	1

Vascularization was assessed subjectively using Colour Doppler, on the epididymal parenchyma in all cases, and on the pampiniform plexus in 7 out of 14 cases. In dogs with acute inflammation, epididymal and pampiniform plexus blood flow appeared increased relative to the contralateral side in unilateral presentations. No appreciable difference in vascularity was noted between epididymis in chronic cases.

**Figure 4:**
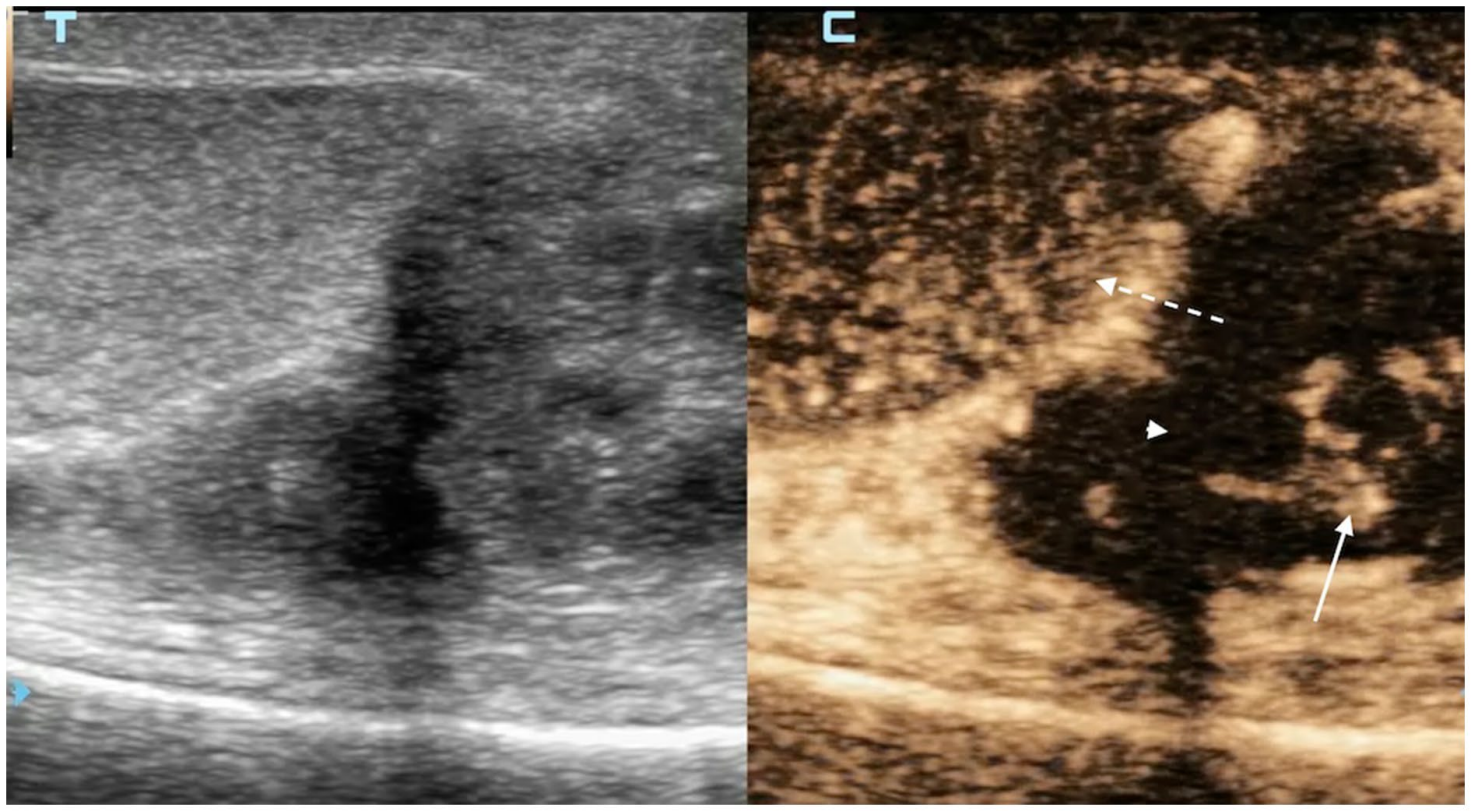
CEUS examination of testis (white dashed arrow) and epididymis affected by chronic epididymitis at peak enhancement after contrast injection. The epididymal parenchyma shows heterogeneous enhancement with avascular areas (white arrowhead), consistent with necrosis confirmed histopathologically, and tortuous parenchymal vessels (white solid arrow).

CEUS was performed in two acute cases and one chronic case, showing higher and inhomogeneous contrast enhancement, of the affected epididymis, but similar time to peak compared to the contralateral side, in both forms. Specifically, the present technique enabled to identify avascular areas corresponding to necrotic tissue, which were not detected using conventional B-mode ultrasound but were confirmed histologically (Figure 4). No specific difference was observed between acute and chronic cases evaluated.

In all cases, both acute and chronic, the ipsilateral testicle showed irregular margins, small mineralized foci and inhomogeneous echotexture. In addition, one case exhibited a testicular abscess, confirmed by histopathology.

**Figure 5:**
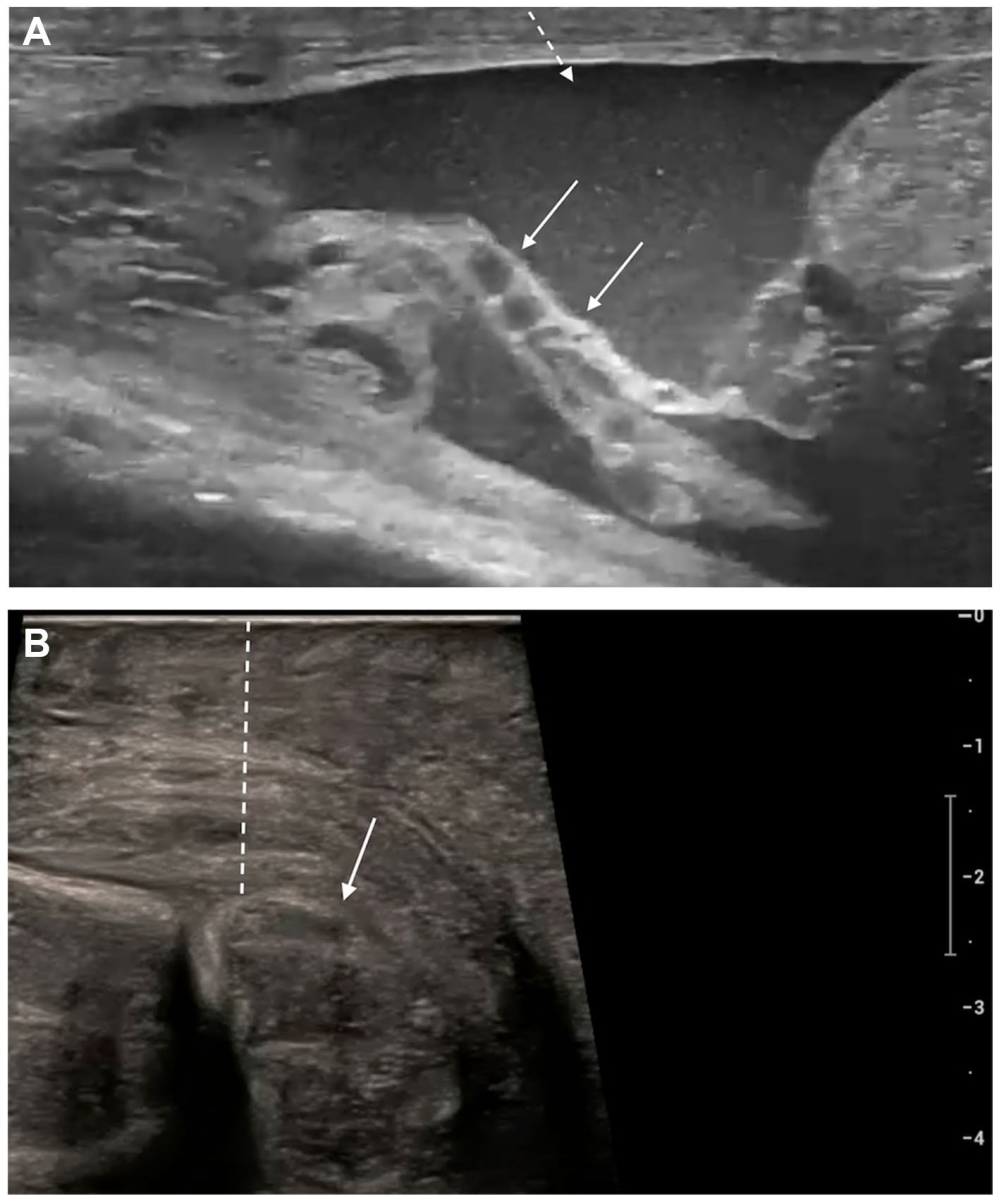
**(A)** Longitudinal view of ductus deferens inflammation (white solid arrows), characterized by increased thickness, endoluminal fluid, hyperechogenicity of the vaginal tunic, and echogenic scrotal effusion (white dashed arrow). **(B)** Longitudinal view of acute orchi-epididymitis (white solid arrow), showing scrotal wall thickening measuring almost 2 cm (white dashed line).

Thirteen of the fourteen cases were presented with concurrent signs of urogenital tract inflammation or infection, including prostatitis, funiculitis and cystitis, whose findings are reported in Table 4. Notably, funiculitis was observed in six cases, being characterized by increased diameter of the ductus deferens, presence of endoluminal fluid, and increased echogenicity of the inguinal and peri-testicular fat, suggestive of steatitis (Figure 5A). Scrotal thickening (Figure 5B), localized pelvic peritonitis and reactivity of the iliac lymph nodes, showing increased size and vascular blood flow, but regular shape, margins and echotexture, were also observed.

**Table 4:** Summary of the ultrasonographic extra-epididymal findings observed in the present population.

Ultrasonographic sign	Number of cases (*n =* 14)	Acute (*n =* 10)	Chronic (*n =* 4)
Testicular abnormalities	14	10	4
Ductus deferens involvement	6	5	1
Reactive iliac lymph nodes	8	6	2
Scrotal thickening	4	3	1

### 3.4 Blood analysis, macroscopical and histopathological findings

Blood analysis indicated inflammatory changes in all acute cases, including leucocytosis, and increased number of segmented and band neutrophiles, and monocytes. In five acute cases, mild to moderate anaemia was observed. Platelet abnormalities were noted in four acute cases, i.e., in three cases thrombocytosis and in one case thrombocytopenia. Three of the four chronic cases showed no remarkable changes in blood values. In contrast, one chronic case presented with elevated leukocytes and band neutrophils, attributable to a concomitant acute cystitis. Elevated alkaline phosphatase was observed in 11 of the 14 cases, including 10 acute and one chronic case. No other specific changes were detected in biochemistry profiles. Findings from blood analysis of the sample population are summarized in Table 5.

**Table 5:** Summary of the blood analysis findings observed in the present population.

Blood analysis findings	Number of cases (*n =* 14)	Acute (*n =* 10)	Chronic (*n =* 4)
Leucocytosis	11	10	1
Band neutrophiles increase	11	10	1
Monocytosis	10	10	0
Anaemia (mild–moderate)	5	5	0
Platelet abnormalities	4	4	0
Alkaline Phosphatase increase	11	10	1

**Figure 6:**
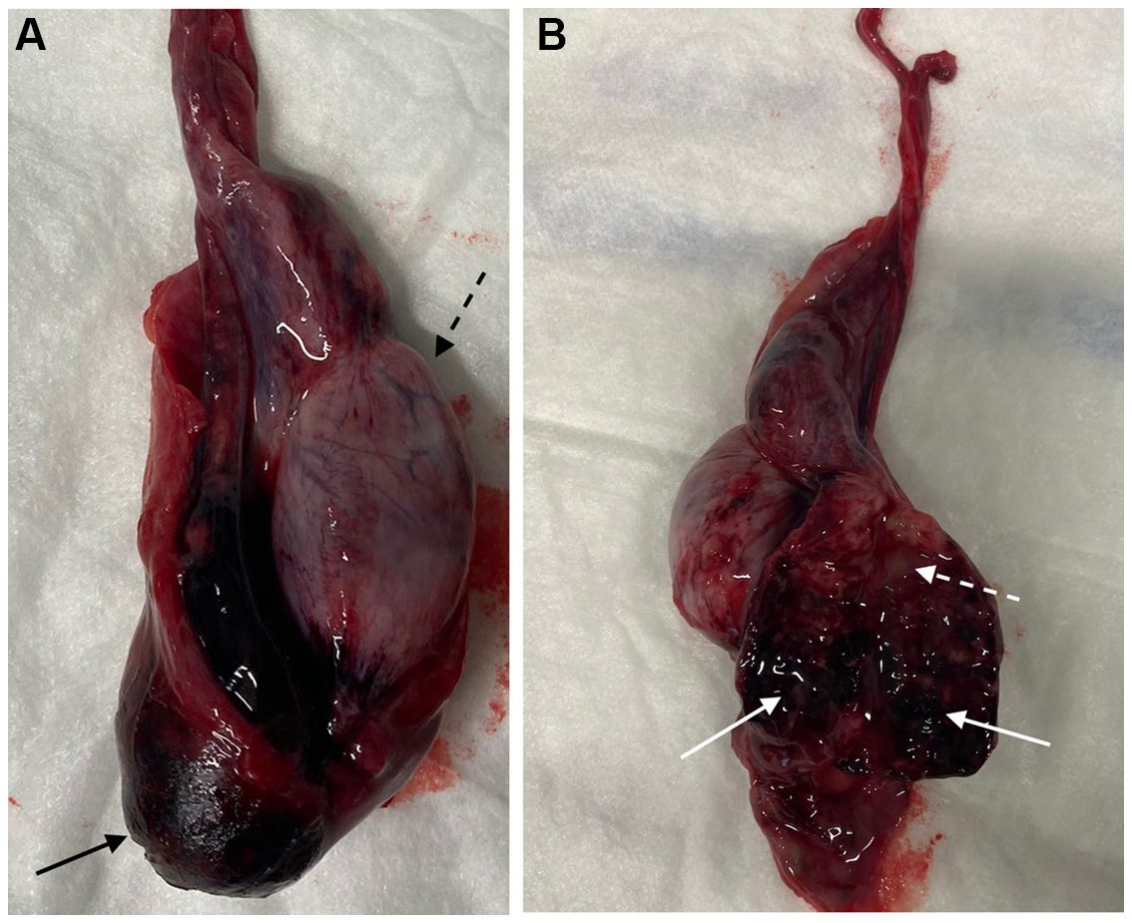
**(A)** Macroscopic examination of necrotizing epididymitis involving head, body, and tail, with hemorrhagic infarction of the tail (black solid arrow), which is nearly the same size as the testis (black dashed arrow). **(B)** Cut surface of the epididymis showing irregular parenchyma with hemorrhagic clots (white solid arrows) and purulent exudate filling multiple cavities (white dashed arrow).

Cases with acute epididymitis exhibited macroscopically a markedly irregular enlarged epididymis, interspersed with hemorrhages and accumulation of yellowish or brownish purulent secretion in cavities or within the parenchyma (Figure 6).

Histopathological examination after orchidectomy was performed in seven dogs: five with acute epididymitis and two with a chronic form. All acute cases exhibited severe, acute, multifocal, purulent necrotizing inflammation of the epididymis with presence of bacteria. In one chronic case evaluated histologically, a high-grade granulomatous epididymitis was diagnosed, characterized by reactive histiocytosis. This lesion included a diffuse infiltrate consisting of numerous histiocytes mixed with fewer small lymphocytes, plasma cells and occasional neutrophils, with focal necrotic areas. No evidence of fungal, bacterial or protozoal pathogens was identified. In the other chronic case, an epididymal abscess and granulomatous inflammation were found.

### 3.5 Treatment

Four dogs with acute epididymitis received medical treatment consisting of antimicrobials (ampicillin, amoxicillin, enrofloxacin) and anti-inflammatory therapy with NSAID, which led to complete remission of clinical signs. In two of these dogs, follow-up ultrasound demonstrated a reduction in epididymal size, heterogeneous appearance of the parenchyma due to the presence of multiple hyperechoic stippling consistent with transition to a chronic condition, within 1 month after treatment. Five dogs with acute epididymitis and two chronic cases underwent bilateral surgical castration. One dog with acute epididymitis died due to unrelated clinical conditions. The remaining two chronic cases did not receive any treatment, as they were completely asymptomatic.

## 4 Discussion

This is the first documented case series on canine epididymitis, integrating ultrasonographic and clinical data. Altogether, ultrasonography proved to be a valuable tool in identifying both acute and chronic forms of the condition. Information on ultrasonographic findings remains limited in literature, with only few single case reports available (1, 4, 5). Some reviews and textbooks also provide description of epididymitis features (7, 13, 16, 19), but in most cases it remains unclear which clinical data or case studies these recommendations are based on. The lack of scientific data is likely related to the low prevalence of the disease, leading to a reduced documentation, especially of the chronic form.

In our case study, we identified a higher proportion of acute (*n =* 10) compared to chronic (*n =* 4) cases. The differentiation represented a major challenge in the present study, as no standardized veterinary criteria are currently available. Moreover, the lack of histopathological examination in all cases may have limited the accuracy of our classification. However, besides histopathology primarily highlights signs of chronicity, we believe that the comprehensive use of clinical, bloodwork and ultrasonographic findings may have consistently reduced the bias. However, the timeframe required for histological features of chronicity to develop in dogs remains unknown. In human medicine, the differentiation is largely based on the duration of clinical signs, supported by imaging, with acute epididymitis defined as a condition lasting less than 6 weeks (20). Following a similar approach, we considered canine cases acute when recent onset of clinical signs suggestive of active inflammation were observed, supported by ultrasonographic evidence and haematological changes. For this case series, we adopted a two-week threshold, in line with recent evidence showing that blood biomarkers of acute inflammation in dogs typically decline within this period (21).

The higher number of acute cases over chronic ones is likely due to the more prominent clinical signs of the first prompting veterinary evaluation, in contrast with the latter often remaining unnoticed. Indeed, dogs with chronic epididymitis were mostly asymptomatic or showing just non-painful scrotal swelling. Hence, owners may not even recognize chronic inflammation and, therefore, do not present their dogs for examination.

The unilateral nature of the disease has been reported also in men, as 96% of the cases occur unilaterally (22). Our data suggests that in dogs more cases affect the left side. To the best of our knowledge, a predisposition of the left or right epididymis to inflammation has never been reported in any species. Nevertheless, the vascular anatomical difference existing between the right and left venous drainage may be a potential explanation, since the right testicular vein drains into the caudal vena cava at the point where its arterial counterpart originates, whereas the left testicular vein carries blood to the left renal vein (16). This anatomical disparity may result in higher hydrostatic pressure and stasis on the left epididymis, predisposing this side to infection and inflammation. However, more research is needed to confirm this hypothesis.

The clinical findings reported in the present case series were already documented in literature, such as painful scrotal swelling and hyperthermia (1, 4, 5). Fever was also reported in the past (23), even though with a lower prevalence.

Concerning ultrasonographic examination, epididymal enlargement was the most consistent finding, especially in acute cases. Pugh et al. (12) provided the only available reference parameters for epididymal size in normal dogs, which were used as thresholds in the present study. However, unfortunately these values were derived from the examination of a small number of individuals and did not take into account potential variations related to age, body weight or fertility status. Establishing well-defined reference values in canine andrology remains essential to improve the standardization of ultrasonographic examinations and the interpretation of reproductive tract findings. In human medicine, the European Academy of Andrology has already provided reference parameters for ultrasonographic measurements of the testes and epididymis (24), underscoring the importance of similar efforts in veterinary medicine.

Capsular irregularity and inhomogeneous echotexture were present in acute and chronic forms, making them nonspecific in the differentiation but supportive findings for the diagnosis of the inflammation. Hyperechoic stippling, cysts, and mineralization were frequent features in acute cases and should raise suspicion for ongoing or past inflammation. However, one of the chronic cases showed an abscess with necrotic tissue, as also reported in a previous report (25). The presence of peri-testicular or -epididymal oedema and scrotal effusion, though less common, may aid confirming acute inflammation, as also described in the past (1, 4, 6).

Unfortunately, to the best of our knowledge, there are no detailed reports describing the clinical and ultrasonographic features of chronic epididymitis in dogs, aside from scrotal swelling, which may persist even 1 year after the initial infection (25). Chronic epididymitis exhibited a variable presentation, ranging from a reduced epididymal size to overt swelling. Importantly, none of these cases showed other clinical or haematological signs indicative of active inflammation. Ultrasound examination consistently revealed hyperechoic stippling of the epididymal parenchyma, suggestive of fibrotic changes, and in two cases, tissue necrosis was also detected. Follow-up of two dogs with acute-onset epididymitis treated with antibiotics showed that, 1 month after therapy, the epididymides had returned to normal size, exhibiting only mild fibrotic changes in the parenchyma. Anyhow, it cannot be excluded that the degree of fibrosis may vary depending on the severity and extent of the initial inflammation. Furthermore, the absence of active inflammation or the presence of reduced epididymal size does not eliminate the potential risk of future inflammatory episodes.

Although no cases of chronic epididymitis progressing into acute disease have been documented in the literature, one report described abscess persistence in a chronic case, which may represent a potential risk for acute exacerbation (25). Necrotic tissue may also serve as a bacterial substrate in the context of ascending infections from other urogenital organs. Therefore, in asymptomatic chronic epididymitis, thorough and repeated reproductive examinations are recommended, especially in older patients or those with coexisting urogenital conditions.

Interestingly, 13 out of 14 dogs presented concurrent pathology of the lower urogenital tract. This finding underlines the potential for bacteria to spread between the bladder, prostate, ductus deferens, epididymis and testicle, suggesting that epididymitis may reflect the progression of other underlying urogenital conditions. The hypothesis that epididymitis is mainly due to ascending infections from the urethra, prostate and urinary bladder is in line with our findings, since the epidydimal tail was the most commonly affected portion, even though in the majority of the cases the entire epididymis was involved in the infection. The tail is the part of the epididymis directly connected to the ductus deferens. Therefore, it is plausible that if an infection ascends via the ductus deferens, the tail would be the first affected portion, underscoring the need to scan all three components. The involvement of the ductus deferens in six cases, together with the involvement of inguinal and pelvic peritoneal fat reinforces this hypothesis. Moreover, this finding aligns with previous information available also in human medicine (22). Iliac lymph nodes were also frequently affected in the present case collection, representing potential sentinels for testicular diseases.

Testicular changes, such as inhomogeneity, mineralization and capsular irregularity, were also present in all cases, reflecting concurrent inflammation. This finding reflects the tight anatomical and functional connection between testicles and epididymis. In a case collection by Camargo-Castaneda et al. (23), it was shown that 3/8 cases of epididymitis had concurrent orchitis and that testicles frequently had degenerative changes within the seminiferous tubules. Degenerative changes can lead to testicular microlithiasis, that has been poorly described in dogs and that is characterized by hyperechoic non-shadowing spots within the testicular parenchyma (16).

In the present case series, the assessment of vascular blood flow was performed in some dogs, showing potential application of other ultrasonographic techniques such as Colour Doppler or CEUS. Colour Doppler showed increased vascularization in acute epididymitis, particularly in the ipsilateral pampiniform plexus, when compared to chronic forms or normal epididymis. This finding aligns with patterns seen in human medicine, supporting the use of this technique especially in terms of comparing the findings between normal and pathological side (26). Nevertheless, the assessment remains prone to subjectivity to a certain extent because no standardized measurement was possible. However, in some cases CEUS could be used to identify avascular necrotic areas not visible on B-mode and may support diagnosis and help formulation prognosis and therapy recommendations. Interestingly, a similar wash-in phase was observed in both acute and chronic cases. This finding might be explained by the time required for acute epididymitis to progress into a chronic condition, as well as by the duration needed for fibrotic processes to develop and subsequently reduce vascular blood supply.

Blood analysis in all acute cases revealed a typical acute inflammatory response, characterized by leucocytosis, neutrophilia, and monocytosis, supporting systemic involvement, consistent with findings reported in previous case reports (1, 5, 6). Interestingly, 11 out of 14 cases showed elevated alkaline phosphatase, which, although nonspecific, may be associated with inflammation.

Interestingly, medical therapy with antibiotics and anti-inflammatory drugs was successful in selected acute cases, representing a potential alternative choice to surgical intervention, but requiring long recovery and treatment timing, especially if preservation of fertility is desired (7). As discussed above potential recurrence, chronicity, and acute relapse of the chronic forms may not be excluded, warranting monitoring. However, the resolution and treatment of the disease is beyond the scope of the present study, especially due to the low number of cases medically treated.

This case series has several limitations, including its retrospective design, which may introduce selection bias due to incomplete records and variability in operator expertise. In addition, it should be considered that the present case population may represent more severe forms of the disease, since dogs were presented to university clinics which may be considered third level services. Moreover, the small sample size limits the possibility to generalize the findings, even though most results are in line with previously published case reports. It should be pointed out that the low sample size is also directly correlated to the low prevalence or limited detection rate of the disease. Further prospective studies comprehensive of follow up and histopathology are needed to confirm the results and standardize ultrasound protocols for epididymal evaluation.

## 5 Conclusion

This case series provides a detailed ultrasonographic characterization of canine epididymitis, correlating our findings with clinical data and highlighting key differences between acute and chronic presentations. Despite limitations, these findings contribute to improving diagnostic accuracy and guiding treatment decisions in dogs with epididymitis.

## Data Availability

The raw data supporting the conclusions of this article will be made available by the authors, without undue reservation.
